# ﻿A fossil species found living off southern California, with notes on the genus *Cymatioa* (Mollusca, Bivalvia, Galeommatoidea)

**DOI:** 10.3897/zookeys.1128.95139

**Published:** 2022-11-07

**Authors:** Paul Valentich-Scott, Jeffrey H. R. Goddard

**Affiliations:** 1 Santa Barbara Museum of Natural History, 2559 Puesta del Sol, Santa Barbara, California 93105, USA Santa Barbara Museum of Natural History Santa Barbara United States of America; 2 Marine Science Institute, University of California, Santa Barbara, Santa Barbara, California 93106, USA University of California Santa Barbara United States of America

**Keywords:** Commensal, intertidal zone, Pleistocene, taxonomy

## Abstract

A small bivalve mollusk previously only known from the Pleistocene of Los Angeles County has recently been found living intertidally near Santa Barbara, California. The bivalve has been determined to be *Cymatioacooki* (Willett, 1937), a member of the Galeommatoidea J.E. Gray, 1840. We document the habitat for the newly discovered *C.cooki*, and compare it to *C.electilis* (Berry, 1963), the other extant member of this genus recorded from the region. *Cymatioacooki* is rare, and while many galeommatoid species have been shown to be commensal with other invertebrates, we have been unable to determine any specific commensal relationships for it.

## ﻿Introduction

The invertebrates inhabiting the rocky intertidal zone of southern and central California are among the most studied and documented in the world (Morris et al. 1980; Ricketts et al. 1985; Carlton 2007). The bivalve mollusks of this region and in this habitat have also been extensively researched (Coan et al. 2000; Coan and Valentich-Scott 2007). It is thus a surprise that a bivalve previously known only from the Pleistocene has been recently discovered living on the underside of intertidal rocks at Naples Point in Santa Barbara County, California. This small, translucent bivalve is clearly identifiable as a member of the frequently cryptic yet exceedingly diverse superfamily Galeommatoidea J.E. Gray, 1840.

Our recently collected specimens belong to the poorly understood genus *Cymatioa* Berry, 1964. The only other living representative of this genus in southern California is *C.electilis* (Berry, 1964). We examined the type specimens of *C.electilis* and concluded they were not the same as our Naples Point species. With subsequent research, we determined our species matched the holotype of *C.cooki* (Willett, 1937) from the Baldwin Hills Pleistocene of Los Angeles County.

Galeommatoidean bivalve mollusks have been extensively documented for nearly 200 years (Turton 1825; Deshayes 1856; Morton and Scott 1989; Goto et al. 2012; Li et al. 2012). Members of the superfamily are exceptionally diverse, with both free-living and commensal species (Li et al. 2016). Those with commensal relationships have been documented living in association with many different invertebrate hosts, including echinoderms, crustaceans, and annelids (Morton and Scott 1989; Goto et al. 2012).

Willett (1937) documented the molluscan fauna at Baldwin Hills, central Los Angeles, during the time a sewer line was being installed. The sewer trench uncovered a 20–30 cm thick Pleistocene deposit of invertebrate and vertebrate fossils, approximately four feet below ground level. In his publication, Willett recognized 296 species of mollusks and described two new species of galeommatoidean bivalves, *Rochefortiareyana* and *Borniacooki* [now *Cymatioacooki*].

Bandy and Marincovich (1973) estimated the Baldwin Hills deposits to be between 36,000 and 28,000 years before the present. The deposits range from 78 to 146 m above current sea level and are approximately 10 km from the modern coastline.

The environment at Naples Point was described in detail by Sousa (1979), who conducted ecological research there (termed the Ellwood Boulder Field) and by Goddard et al. (2020), who conducted a long-term study of heterobranch sea slugs at the point. Common macro-invertebrates observed under boulders and cobbles by the latter included Striped Shore Crabs *Pachygrapsuscrassipes* Randall, 1840, juvenile Bat Stars *Patiriaminiata* (Brandt, 1835), juvenile Purple Sea Urchins *Strongylocentrotuspurpuratus* (Stimpson, 1857), the Banded Turban Snail *Tegula eiseni* Jordan, 1936, the chitons *Stenoplaxconspicua* (Dall, 1879), *Lepidozonapectinulata* (Carpenter in Pilsbry 1893), and *Leptochitonrugatus* (Carpenter in Pilsbry 1892), the Tidepool Ghost Shrimp *Neotrypaeabiffari* (Holthuis, 1991) and its commensal goby *Typhlogobiuscaliforniensis* (Steindachner, 1879), the Peanut Worm *Phascolosomaagassizii* Keferstein, 1866, the brittle star *Ophioplocusesmarki* Lyman, 1874, and juvenile two-spot octopuses (*Octopus* sp.). It was near the end of the study by Goddard et al. (2020) that he found the living galeommatid bivalve described herein.

## ﻿Materials, methods, site details, and abbreviations

**Figure 1.**
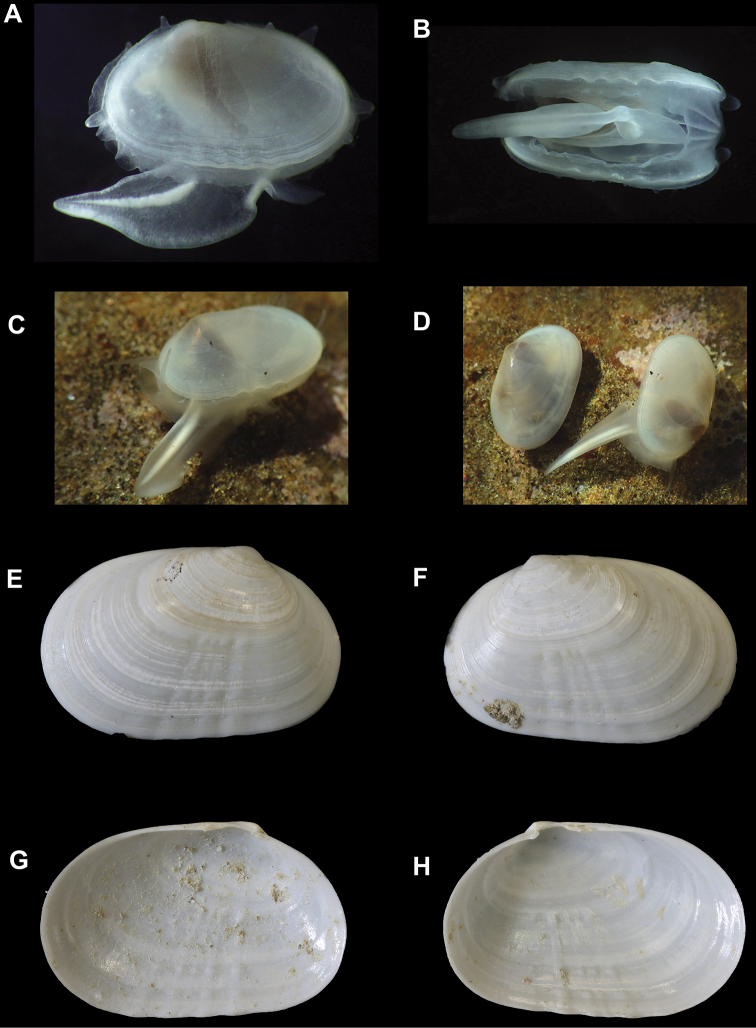
*Cymatioacooki*. **A, B** living animal from Naples Point, SBMNH 629938, length = 7.4 mm **A** lateral view with extended foot, note mantle papillae anteriorly and dorsally **B** ventral view with wide, long mantle gape **C, D** animals on native substratum **E–H** holotype, LACMIP 59.2., length = 9.7 mm **E** exterior of right valve **F** exterior of left valve **G** interior of left valve **H** interior of right valve.

Abbreviations: **CASIZG**, Invertebrate Zoology and Geology, California Academy of Sciences, San Francisco, California, USA; **SBMNH**, Invertebrate Zoology, Santa Barbara Museum of Natural History, Santa Barbara, California, USA; **LACMIP**, Invertebrate Paleontology, Natural History Museum of Los Angeles County, Los Angeles, California, USA.

**Figure 2.**
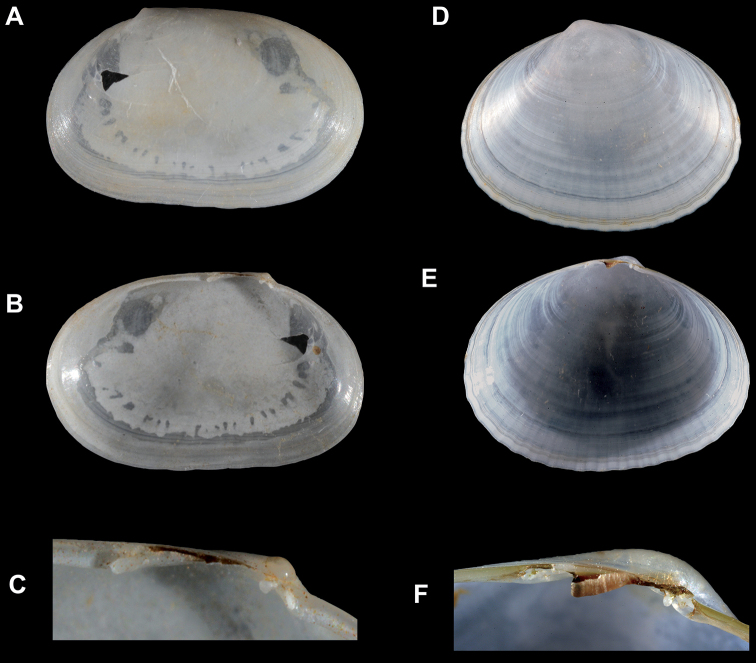
**A–C***Cymatioacooki*, shell of left valve collected at Naples Point, SBMNH 641848, length 8.8 mm **A** exterior of valve **B** interior of valve **C** close up of hinge **D–F***Cymatioaelectilis*, left valve **D, E** holotype, CASIZG 043976, length = 16 mm **D** exterior of valve **E** interior of valve **F** paratype, SBMNH 34017, close up of hinge.

The galeommatid bivalve we describe here was collected by hand by the second author at Naples Point, located on the south coast of Santa Barbara County, 24 km west of Santa Barbara (approximately 34.43, -119.95). This area is within the Naples State Marine Conservation Area. The second author also found two living specimens, shell length about 10 mm, on 23 November 2018, under a low intertidal boulder and photographed but did not collect them (Fig. 1C, D). On 4 March 2019, the second author found a third specimen, shell length 7.4 mm, on the underside of a low intertidal boulder, about 10 m east from where the first two specimens were found. After the third specimen was photographed *in situ* and collected, additional images were taken following relaxation in MgCl_2_ (Fig. 1A, B). On 10 December 2019, a fourth specimen, a left shell valve 8.8 mm long, was found underneath a low intertidal boulder (Fig. 2A–C). Subsequent visits to the same locality did not yield any additional shells or living animals.

## ﻿Systematic account

**Superfamily Galeommatoidea J.E. Gray, 1840**

**Family Galeommatidae J.E. Gray, 1840**

### ﻿ Cymatioa

Taxon classificationAnimaliaGaleommatidaGaleommatidae

Berry, 1964

1A2DD850-D91B-5498-9C0C-80475498D2A9

Crenimargo Berry, 1963, not Cossmann, 1902. Type species (monotypy): Crenimargoelectilis Berry, 1963. Recent, eastern Pacific.
Cymatioa Berry, 1964, new name for Crenimargo Berry, not Cossmann.

#### Description.

Shell ovate; subequilateral; exterior surface finely punctate; sculpture of sparse, broad, low, radial ribs; ventral margin undulate; right valve with one anterior cardinal tooth; left valve with two anterior cardinal teeth.

#### Commensal relationships.

Baldwin (1990) reported *Cymatioaelectilis* from Nayarit, Mexico, 20 cm deep and byssally attached to the walls of the burrows of the ghost shrimp, *Axiopsisserratifrons* (Milne-Edwards, 1873).

#### Discussion.

Huber (2015) suggested a number of species that might fall within *Cymatioa* based on their punctate sculpture and undulate ventral margin. While the type species of *Cymatioa* was described from Colima, Mexico, the species he included in this genus are distributed in tropical locations around the globe.

### ﻿ Cymatioa cooki

Taxon classificationAnimaliaGaleommatidaGaleommatidae

(Willett, 1937)

AAEEBCC5-9842-5D91-81C5-B50317A2882E

Figs 1A–H , 2A–C

Bornia cooki Willett, 1937: 389, pl. 5, figs 3–6.

#### Description.

***Shell***: thin, fragile, subovate; inequilateral, posterior end much longer; anterior and posterior ends broadly rounded; dorsal margin gently sloping on each side of umbos; ventral margin broadly gaping in living animal; beaks small, sharply pointed; prodissoconch 200 µm in diameter; sculpture of irregular, slightly wavy commarginal striae, and fine, dense punctae; ventral margin with sparse, broad, low radial undulations; periostracum thin, light beige, silky; hinge plate narrow; right valve with one short anterior cardinal tooth, one elongate posterior lateral tooth; left valve with two minute anterior cardinal teeth, one elongate posterior lateral tooth; ligament internal, opisthodetic, elongate; resilifer narrow, elongate; ventral margin slightly wavy internally; adductor muscle scars subovate, subequal; pallial line entire; strong accessory muscle scars dorsal to pallial line. Length to 11.4 mm (Willett 1937).

***Mantle***: large, reflected, covering most of outer shell surface when fully extended, including umbones (Fig. 1A); mantle can be mostly retracted into the shell; reflected portion of mantle sparsely papillate (Fig. 1A); slightly fused posteroventrally; two anterior and two posterior tentacles, short, slightly extending past shell margins (Fig. 1A, B).

***Foot***: large, translucent, exceeding the length of the shell when fully extended, spathate, with distinct pointed heel; bright white stripe extending from the tip of foot to the shell margin, presumably related to byssal formation (Fig. 1A). This species is an active crawler (Fig. 1C).

#### Type locality.

Baldwin Hills Pleistocene deposit, Los Angeles County, California; 33.9658, -118.4264; LACMIP locality 59.

#### Locality of living specimens.

USA, California, Santa Barbara County, off Naples Point; 34.4339, -119.9500; intertidal zone, in boulders and cobbles. SBMNH 629938, conjoined shell and anatomy, length 7.4 mm, height 4.5 mm (Fig. 1A, B); SBMNH 641848, (Fig. 2A–C), one left valve length 8.8 mm, height 5.5 mm.

#### Habitat and potential commensal relationships.

All three living specimens were found near the seaward edge of a boulder field centered at 34.4339, -119.9500 and located on a broad, gently sloping, wave-cut bench of Monterey Shale. This boulder field extends vertically from a tidal height of approximately +0.3 m above mean lower low water to −0.4 m. The surfgrass *Phyllospadixtorreyi* S. Watson, 1879, dominates much of the surrounding bench. At low tide, a shallow lagoon lies just landward of the boulder field, and behind that are more shale bench, a narrow sand beach, and then cliffs up to 20 m high consisting of Monterey shale overlain by terrestrial deposits. Sand levels on the beach and in the lagoon fluctuate seasonally, with nearly all of the beach scoured away in winter, but the boulder field as a whole is never significantly inundated, especially at its seaward edge where the *Cymatioa* was found. Vertical relief in the boulder field is fairly low, with most boulders under 0.5 m diameter. A few rock outcrops just to the west are only about 1 m high.

The specimens found on 23 November 2018 were on sand underneath a boulder (Fig. 1C, D). One of these was found at the entrance to a burrow of unknown origin, with its foot extended and tentaculate inhalant siphon extending into the burrow opening. The burrow may have been constructed by the Tidepool Ghost Shrimp, *Neotrypaeabiffari* (Holthuis, 1991), which occur frequently under boulders at this site, usually with commensal Blind Gobies *Typhlogobiuscaliforniensis* Steindachner, 1879. This sighting is vouchered in eight images at https://www.inaturalist.org/observations/18597683, with the last image showing one of the specimens as first observed, next to the burrow entrance described above.

The specimen found on 4 March 2019 was on the underside of a boulder, among scattered tubes of the annelid *Spirorbis* sp. and small, scattered patches of an unidentified tan-colored encrusting sponge. Two small dorid nudibranchs, *Conualeviaalba* Collier & Farmer, 1964; a single mussel, *Mytiliseptabifurcata* (Conrad, 1837); and an adult chiton, *Stenoplaxconspicua* (Dall, 1879), were also present, all within a few centimeters of the *C.cooki*. Burrow openings of unknown origin and 3–5 mm in diameter were also present on the undersurface of the boulder. This sighting is vouchered in six images at https://www.inaturalist.org/observations/20962245.

#### Comparisons.

The shell morphology of *C.cooki* is closest to *C.electilis*, with both species sharing a commarginal and punctate sculpture and an undulate ventral margin (Fig. 2A–F). *Cymatioacooki* is subquadrate and inequilateral, with a much longer posterior end (Fig. 2A), whereas *C.electilis* is subovate with a slightly longer posterior end (Fig. 2D). The cardinal teeth in both species are quite small and similar; however, the posterior lateral tooth in *C.cooki* is longer and more robust (Fig. 2C) than that of *C.electilis* (Fig. 2F). Because the living animal is undocumented for *C.electilis*, we are unable to provide anatomical comparisons. However, based on other galeommatid taxa, many differences in mantle tentacles and papillae are likely.

## ﻿Discussion

Previously only known from the Pleistocene of Los Angeles, *Cymatioacooki* is herein recorded living for the first time. Only three living specimens have been discovered to date. Despite *C.cooki*’s potential commensal relationship with burrowing invertebrates, we have not sampled the intertidal infauna deeply enough to discover the potential true habitat for this species.

Depending on the lifespan of *C.cooki*, the adults we observed at Naples Point may have been transported as larvae from much farther south during the marine heatwaves of 2014–2016, which drove northward numerous marine species distributions in the northeastern Pacific (Cavole et al. 2016; Sanford et al. 2019), including populations documented specifically at Naples Point (Goddard et al. 2016, 2018). This might explain why the second author did not find *C.cooki* at this site prior to 2018, despite intensively searching the same under-rock habitat for heterobranch sea slugs at Naples Point since 2002 (Goddard et al. 2020).

Other Baldwin Hills Pleistocene bivalves reported by Willett (1937) have been documented as living in southern California. *Mytilusadamsianus* [= *Brachidontiesadamsianus* (Dunker, 1857)] is a common modern rocky intertidal species from Santa Cruz Island, California, to northern Peru (Coan and Valentich-Scott 2012). *Ensiscalifornicus* [= *Ensismyrae* (Berry, 1953)] and *Petricola* “*tellimyalis*” [= *Petricolahertzana* (Coan, 1997)] are also found intertidally in southern California with the former in sandy protected environments and the latter associated with giant kelp holdfasts (Coan and Valentich-Scott 2012). The *Cymatioa* specimen described by Willett (1937) was named for a Miss Edna T. Cook, who collected the specimens.

Given the small size, translucent shell, and cryptic habits of *C.cooki*, it is not surprising that living instances of the species have been overlooked for over 80 years. We are confident that its description here will lead to discovery of further examples in southern California and likely even further south into Mexico.

## Supplementary Material

XML Treatment for Cymatioa

XML Treatment for Cymatioa cooki
